# Maternal priorities for preventive therapy among HIV‐positive pregnant women before and after delivery in South Africa: a best–worst scaling survey

**DOI:** 10.1002/jia2.25143

**Published:** 2018-07-04

**Authors:** Hae‐Young Kim, David W Dowdy, Neil A Martinson, Jonathan E Golub, John F P Bridges, Colleen F Hanrahan

**Affiliations:** ^1^ Department of Epidemiology Johns Hopkins Bloomberg School of Public Health Baltimore MD USA; ^2^ Africa Health Research Institute Durban KwaZulu‐Natal South Africa; ^3^ School of Nursing & Public Health University of KwaZulu‐Natal Durban KwaZulu‐Natal South Africa; ^4^ Department of International Health Johns Hopkins Bloomberg School of Public Health Baltimore MD USA; ^5^ Perinatal HIV Research Unit University of Witwatersrand Johannesburg South Africa; ^6^ Center for Tuberculosis Research Johns Hopkins University Baltimore MD USA; ^7^ Department of Health Policy and Management Johns Hopkins Bloomberg School of Public Health Baltimore MD USA; ^8^ Department of Health Behavior and Society Johns Hopkins Bloomberg School of Public Health Baltimore MD USA

**Keywords:** Pregnant women, preventive therapy, maternal priorities, preferences, HIV, TB, South Africa

## Abstract

**Introduction:**

Pregnant women newly diagnosed with HIV during pregnancy are often lost to follow up and their adherence rates drop after delivery. We quantified changes in priorities related to isoniazid preventive therapy (IPT) and antiretroviral therapy (ART) among pregnant women living with HIV.

**Methods:**

We enrolled pregnant women recently diagnosed with HIV from 14 primary health clinics during pregnancy and followed them after delivery in Matlosana, South Africa. Best–worst scaling (BWS) was used to determine the women's priorities out of 11 attributes related to preventive therapy in the ante‐ versus postpartum periods. Aggregate BWS scores were calculated based on the frequency with which participants selected each attribute as the best or worst among five options (across multiple choice sets). Individual BWS scores were also calculated and rescaled from 0 (always selected as worst) to 10 (always selected as best), and changes in BWS scores in the ante‐ versus postpartum periods were compared, using a paired *t*‐test. Factors associated with the changes in BWS scores were examined in multiple linear regressions. Spearman's rho was used to compare the ranking of attributes.

**Results:**

Out of a total of 204 participants, 154 (75.5%) completed the survey in the postpartum at the median 15 (IQR: 11 to 27) weeks after delivery. Trust in healthcare providers was most highly prioritized both in the ante‐ (*individual BWS Score* = *7.34, SE* = *0.13)* and postpartum periods (*BWS* = *7.21* ± *0.11*), followed by living a long life (*BWS* = *6.77* ± *0.09* in the ante‐ vs. *BWS* = *6.86* ± *0.10* in the postpartum*)*. Prevention for infants’ health was more prioritized in the post‐ (*BWS* = *6.54* ± *0.09*) versus antepartum periods (*BWS* = *6.11* ± *0.10*) (*p* = 0.05). This change was associated with IPT initiation at enrolment (regression coefficient = 0.78 ± 0.33, *p* = 0.001). Difficulty in daily pill‐uptake was significantly more prioritized in the postpartum (*BWS* = *5.03* ± *0.11*) than in the antepartum (*BWS* = *4.43* ± *0.10*) (*p* < 0.01). Transportation cost and worry about side effects of pills were least prioritized. Overall ranking of attributes was similar in both time periods (spearman's rho = 0.90).

**Conclusions:**

Comprehensive interventions to build trust in healthcare providers and support adherence may increase uptake of preventive therapy. Counselling needs to emphasize medication benefits for both maternal and infant health among HIV‐positive pregnant women.

## 1 Introduction

In 2016, there were 10.4 million new cases of tuberculosis (TB) and 1.3 million deaths associated with it worldwide 1. South Africa has some of the highest rates of HIV and TB in the world and over 50% of TB incident cases are co‐infected with HIV. Pregnancy is often the gateway for HIV diagnosis in women of reproductive age, in which HIV seroprevalence was about 30.8% in 2015 2. TB increases maternal mortality and adverse infant outcomes among HIV‐positive women during pregnancy and in the postpartum period 3, 4, 5.

Antiretroviral therapy (ART) and isoniazid preventive therapy (IPT) are two key interventions to reduce TB incidence and ensure better long‐term health outcomes of both HIV‐positive pregnant women and their infants 1, 6. When taken together with ART, IPT can decrease the risk of developing active TB up to 90% among people living with HIV (PLWH) 7, 8. Since January 2015, the South African national guideline recommends immediate initiation of lifelong ART (i.e. Option B+) and IPT up to 36 months for HIV‐positive pregnant women 6. While ≥95% of pregnant women with HIV presented at ANC received ART in 2015 9, up to 50% have been reported as lost to follow‐up (LFU) after delivery 10. However, the implementation of IPT remains poor. The coverage of IPT among PLWH is highly variable, ranging 2% to 73% 1, and over‐reporting of IPT initiation has been reported in South Africa 11.

Pregnant women diagnosed with HIV are often motivated to take ART to prevent HIV transmission during pregnancy but might stop seeking care once they have safely protected their infants from infection 12. Changes in circumstances, such as taking care of a new baby and returning to work, or negative treatment from clinic staff have been reported as factors related to ART discontinuation in the postpartum period 13, 14, 15. Fear of side effects and non‐disclosure of HIV status were also associated with low uptake of IPT among PLWH 16. Yet previous qualitative studies reported that HIV‐positive pregnant women who perceived the benefits of ART as “life‐giving” and received treatment support from healthcare workers could achieve better adherence in the postpartum period 17, 18.

Understanding maternal priorities for preventive therapy is crucial to deliver services in the most acceptable way to these women. BWS is a stated‐preference method which allows to analyse preferences among a set of attributes characterizing goods or services and has been used in over 50 different applications in healthcare or services research 19, 20, 21. In this study, we quantified how HIV‐positive pregnant women evaluate and prioritize different factors related to infant health, their own health and structural challenges regarding uptake of ART and IPT using BWS and examined factors associated with changes in maternal priorities before and after delivery.

## 2 Methods

### 2.1 Study population and setting

We recruited HIV‐positive pregnant women from 14 primary care public health clinics (PHC) in the Dr. Kenneth Kaunda health district in the North West province of South Africa from February 2015 to December 2016. The HIV prevalence among antenatal clinic attendees in Dr. Kenneth Kaunda was 30.9% in 2015 2. These 14 clinics were selected to use the existing study structure of an ongoing cluster randomized trial, which compares the proportion with known TB infection status and IPT initiation among newly diagnosed HIV patients in clinics, using two different diagnostic tests for latent TB infection 22, 23.

Clinics were chosen to cover a range of patient volumes, geographic locations, clinic hours, and urban versus rural settings. Patients were eligible for enrolment if they were ≥18 years old, currently pregnant, diagnosed with HIV in the preceding six months, and able to read either English, Xhosa, Setswana or Zulu since the BWS instrument involved reading choice tasks. The literate rate among adults is 94% 24 and questionnaires were offered in the four languages. We sought to enroll all eligible pregnant patients during the study period. All interviews were conducted in a private room to protect patients’ confidentiality. Written informed consents were obtained from all participants. The study was approved by both the institutional review boards at the Johns Hopkins School of Medicine and the University of Witwatersrand.

### 2.2 Study design

We conducted a longitudinal survey incorporating an object case BWS instrument. All pregnant women were enrolled during pregnancy and scheduled to complete follow‐up visits at 6 and 14 weeks postpartum, aligning with their infants’ routine immunization visits 6. However, most participants only completed either 6 or 14 weeks visit thus only one postpartum visit per respondent was included for the analysis. The 14 weeks visit was included as the primary point; if a participant missed the 14 weeks visit, 6 weeks or later than 14 weeks visit, whichever was closer to the scheduled 14 weeks visit, was included. We conducted sensitivity analyses to examine potential impact of different timing of postpartum visits (See supplement).

To our knowledge, this is the first study to use BWS among HIV‐positive pregnant women. BWS is based on the assumption that a respondent has underlying utility associated with each attribute and can choose the best (most important) and the worst (least important) attribute from a set of available options in a given choice task as a rational choice maker 25, 26, 27, 28. Of three variants of BWS, object case BWS allows to rank the relative importance of attributes in a continuous quantitative scale 21. Recently, various ways have been suggested to test reliability and validity of BWS 21, 29.

### 2.3 Selection of attributes

We identified the attributes most likely to influence women's decision‐making regarding uptake of preventive therapy through literature review and prior in‐depth interviews about patients’ experiences and perceptions of ART and IPT 16, 23, 30, 31. Four themes emerged: treatment benefits for maternal and infant heath, interpersonal support, trust in healthcare services, and structural barriers. A total of 11 attributes were chosen, including roughly equal numbers of potential barriers and facilitators for initiation of IPT (Table 1). Attributes were converted to simple statements, which were then reviewed and refined by clinical experts and healthcare providers in the clinics. Identification and refinement of statements followed the framework for instrument development of a choice experiment 28.

**Table 1:** Selected attributes related to preventive therapies tested in the best–worst scaling task

Attributes	Statements
Trust in healthcare providers for infant's health	I trust that doctors and nurses know what is best for infant's health
Prevention for infant's illness	Medications I take prevent my infant to get infected or become sick
Side effects on infant	I worry that if I take pills, it can cause side effects on my infant
Strength	Medications to prevent disease help me feel stronger
Long life	I can live as long as someone without HIV if I take care of myself
Interpersonal support for pill take	Friends and families help me to take medications
Fear of unintended disclosure of HIV status	I worry that taking pills every day tells other people that I have HIV
Knowledge on pill purpose	I know the purpose of each different medication I take
Difficulty in daily adherence	I have trouble taking medication on a daily basis
Travel cost	Getting to the clinic costs me too much money
Lack of time	I am too busy to come to regular clinic visits

### 2.4 Experimental design

**Figure 1:**
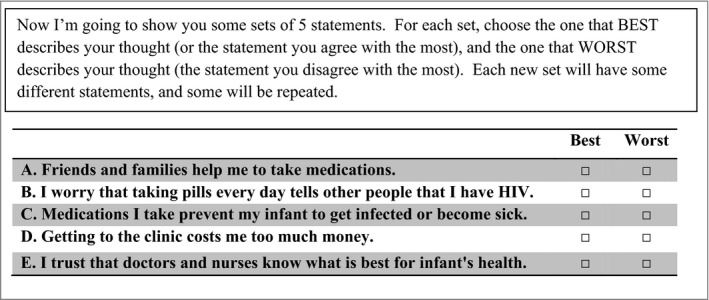
Example of a best–worst scaling choice task with five attributes related to preventive therapies among HIV‐positive pregnant women.

We used a balanced incomplete block design in a main‐effects orthogonal array, which allows for repetition of different combinations of attributes in the subsets of all possible choice scenarios 32. Each respondent was presented with five statements in each given choice task and asked to choose one statement out of the five that best described her thoughts and another statement that worst described her thoughts. Each of the 11 statements appeared five times across 11 separate choice tasks (Figure 1).

### 2.5 Sociodemographic, clinical variables and adherence measurement

Participants’ sociodemographic and clinical information was obtained at enrolment. Details related to delivery and history of breastfeeding were obtained in the postpartum visits. Infant HIV status was recorded if the HIV test results were available. Participants were asked about the degree of support they received from healthcare workers, family or friends with regard to taking medications and whether they were satisfied with that support. Self‐reported adherence rates to ART and IPT were measured by the AIDS Clinical Trials Group (ACTG) Adherence Questionnaire, which is based on a four‐day recall with five items. This questionnaire has been extensively used and validated in other studies 33, 34, 35, 36. Adherence to ART or IPT was defined as taking all drugs in the previous four days. Adherence to IPT was also measured by testing eligible participants’ urine samples by IsoScreen kit (GFC Diagnostics Limited, Oxfordshire, UK).

### 2.6 Data analysis

The outcome was the participants’ choice of best and worst statements in each subset of statements presented to them in the ante‐ and postpartum periods 37. The frequency of being chosen as the best and worst statement across all choice tasks was calculated, resulting in a relative BWS score 38. Each statement chosen as best received a score of 2, while the statement chosen as worst received a score of 0 and all other non‐selected statements a score of 1. Since each statement appeared five times per respondent, the scores for each statement ranged from 0 (always selected as worst) to 10 (always selected as best) for individual BWS scores. The aggregate BWS score was also calculated as the mean score across all respondents, rescaled from 0 to 100. Thus, a mean score of 50 would mean either no selection or neutral selection (i.e. an attribute is chosen as best and worst for the equal number of times). Previous studies have shown that a simple BWS score has good validity, compared to more sophisticated regression‐based methods, including conditional logistic regression 39, and good discrimination compared to rating or ranking scales 40.

The difference in the ante‐ versus postpartum periods was calculated and compared, using a paired t‐test. We examined the potential association between individual BWS scores and clinical factors (CD4 cell counts, IPT initiation, perceived risk of TB in next year and adherence to ART) and sociodemographic factors (transportation cost to the clinic and number of previous pregnancy) in multiple linear regressions. Final multiple linear regression models were fitted adjusting for individual covariates with *p*‐value <0.05. All analyses were conducted in STATA 13.

## 3 Results

### 3.1 Patient characteristics at baseline and follow‐up

Of the 204 pregnant women enrolled, 47 (23%) did not complete the follow‐up visits: 20 participants were lost to follow up, 23 were contacted three times but could not be reached, and 4 lost interest in the study or refused to participate further. The baseline characteristics of participants who completed follow‐up visits did not differ from those lost to follow up.

Of 154 who completed at least one follow‐up visit, 77 (50%) were followed at 14 weeks, 33 (21%) at 6 weeks and 44 (29%) at later than 14 weeks visits. The median time since delivery at follow‐up visits was 15 (IQR: 11 to 27) weeks. At enrolment, approximately 30% (n = 45) were receiving IPT, while 98% (n = 151) were on ART (Table 2). The time from initial HIV diagnosis was slightly longer in those receiving IPT (78 ± 68 days) than in those not receiving IPT at enrolment (45 ± 41 days, *p* < 0.001). About 80% (n = 125) of the participants had disclosed their HIV status to their partners.

**Table 2:** Baseline characteristics by the current status of Isoniazid Preventive Therapy (IPT) among 154 HIV‐positive pregnant women, South Africa

N (%)	Receiving IPT at enrolment (N = 45)	No IPT (N = 109)	*p*‐value^a^
Age (years), Mean (±SD)	28 (±6)	27 (±6)	0.89
Gestational week at first ANC visit, Mean (±SD)	19 (±6)	18 (±8)	0.70
Gestational week at enrolment, Mean (±SD)	27 (±7)	24 (±9)	0.05
Time since HIV diagnosis (days), Mean (±SD)	78 (±68)	45 (±41)	<0.001
CD4 cell count (cells/mm^3^), Mean (±SD)^b^	481 (±227)	467 (±248)	0.79
Perceived risk of developing TB within next year^c^			
Slightly likely	16 (35.6)	12 (11.1)	<0.001
It is not at all likely	29 (64.4)	96 (88.9)	
Education^b^			
≤9th grade	9 (25.0)	21 (26.9)	0.83
>9th grade	27 (75.0)	57 (73.1)	
Employment status			
Full time	2 (4.4)	11 (10.1)	0.35
Part time or piece jobs	5 (11.1)	7 (6.4)	
Unemployed	38 (84.4)	91 (83.5)	
Mode of transportation to clinic			
On foot	23 (51.1)	61 (56.0)	0.86
Public taxi or bus	21 (46.7)	46 (42.2)	
Private car or motorbike	1 (2.2)	2 (1.8)	
Transportation cost (Rand)			
Median (IQR)	0 (0, 20)	0 (0, 16)	0.14
Marital status			
Married	7 (18.4)	10 (9.3)	0.31
Living with partner	13 (34.2)	39 (36.1)	
Not living with partner	18 (47.4)	59 (54.6)	
Disclosure of HIV status to partner			
Yes	28 (62.2)	54 (50.5)	0.18
No	17 (37.8)	53 (49.5)	

a Pearson χ^2^ test (discrete variables), *t*‐test (mean comparison for continuous variables) and Mann–Whitney test (median comparison for continuous variables) were used.

b Some variables have missing data.

c No one chose other categories of *moderately likely, very likely or extremely*.

Two (1.3%) infants were found to be HIV‐positive, 125 (89.3%) HIV‐negative and 13 (8.4%) had unknown HIV status. HIV test results were not available for 10 (6.5%) infants. Nearly 90% (n = 132) of participants had ever breastfed their infants, and 57% (n = 88) were still breastfeeding at the time of follow‐up including 52 women having exclusively breastfed. While 91% (n = 139) reported they received some or a lot of support from healthcare providers to remind them about taking medications, 47% (n = 72) got this from friends and family (*p* < 0.001), and 21% (n = 32) were somewhat or very dissatisfied with treatment support received from friends and family. Ninety‐five percent (142/150) of patients on ART reported that they were informed about the side effects and the reasons to take it compared to 72% (23/32) of people on IPT.

### 3.2 Adherence to ART and IPT

Everyone was on ART in the postpartum visits. Over 90% (140/149) were fully adherent to ART. Of 36 initiated on IPT prior to enrolment, 14 were still prescribed to take IPT in the postpartum period, and all participants (n = 10) who completed the adherence questionnaire self‐reported as fully adherent but 70% were considered as adherent when tested by the IsoScreen test. Among 18 participants initiated on IPT after enrolment, 14 completed the adherence questionnaires: 13 (93%) self‐reported as adherent and 9 (64%) were considered as adherent by IsoScreen test.

### 3.3 Aggregate BWS scores in the antepartum and postpartum periods

Trust in healthcare providers was prioritized most highly in the postpartum period (*BWS* = *72.1, SE* = *2.6*), followed by having a long life (*BWS* = *68.6 *±* 2.5*). This did not differ significantly from the antepartum period (Table 3). Prevention of infant illness had significantly higher scores in the postpartum period (*BWS* = *65.4 *±* 2.4)* than in the antepartum period (*BWS* = *61.1 *±* 2.2*) (*p* < *0.01)*. Travel costs and side effects for infants were less prioritized in the postpartum period.

**Table 3:** Frequency of attributes chosen among 11 statements and aggregate BWS scores in the antepartum versus postpartum (N = 154)

11 Statements	Antepartum				Postpartum				*p*‐value
	Best^a^	Worst^b^	BWS score	SE	Best	Worst	BWS score	SE	
I trust that doctors and nurses know what is best for infant's health.	387	27	73.4	2.6	361	20	72.1	2.6	0.38
I can live as long as someone without HIV if I take care of myself.	324	51	67.7	2.4	306	19	68.6	2.5	0.53
Medications I take prevent my infant to get infected or become sick.	241	70	61.1	2.2	265	28	65.4	2.4	<0.01
Medications to prevent disease help me feel stronger.	213	45	60.9	2.2	148	20	58.3	2.1	0.04
I know the purpose of each different medication I take.	201	34	60.8	2.2	227	19	63.5	2.3	0.04
I have trouble taking medication on a daily basis.	70	157	44.3	1.6	104	100	50.3	1.8	<0.001
Friends and families help me to take medications.	117	243	41.7	1.5	98	266	38.9	1.4	0.1
I am too busy to come to regular clinic visits.	45	197	40.1	1.4	69	154	44.5	1.6	<0.01
Getting to the clinic costs me too much money.	25	195	39.0	1.4	16	244	35.2	1.3	<0.01
I worry that if I take pills it can cause side effects on my infant.	38	290	33.6	1.2	18	350	28.4	1.0	<0.001
I worry that taking pills every day tells other people that I have HIV.	27	376	27.3	1.0	43	435	24.5	0.9	0.06

a Number of times chosen as best attribute in a given choice set is calculated. A total of 770 (154 respondents × 5 times per attribute) was available to be selected per attribute.

b Number of times chosen as worst attribute in a given choice set is calculated.

### 3.4 Individual BWS scores in the antepartum and postpartum periods

**Figure 2:**
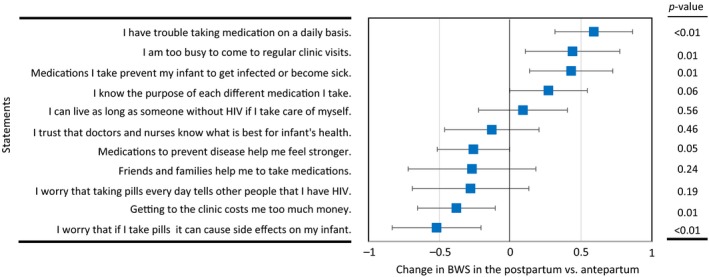
Changes in individual best–worst scaling (BWS) scores for 11 statements related to preventive therapies among HIV‐positive pregnant women in the postpartum versus antepartum periods. Error bars show 95% confidence intervals, and *p*‐values from Wald tests comparing mean BWS scores in the postpartum versus antepartum periods are presented to the right.

When individual BWS scores were compared in the antepartum versus postpartum periods, we observed similar patterns of prioritization (Table 4). Trust in healthcare providers for infants’ health was prioritized most highly both in the ante‐ (*BWS* = *7.34, SE* = *0.13*) and postpartum periods (*BWS* = *7.21 *±* 0.11*), and these did not significantly differ (*p* = 0.46) (Figure 2). Having a long life was similarly prioritized in the ante‐ (*BWS* = *6.77 *±* 0.13*) and postpartum periods (*BWS* = *6.86 *±* 0.10*) (*p* = 0.56). Compared to the antepartum period, prevention of infant illness was slightly more prioritized in the postpartum period (*p* = 0.05) as well as difficulty in taking pills daily (*p* < 0.01) and lack of time to make regular clinic visits (*p* = 0.01). Fear of unintended disclosure of HIV status was least prioritized in both antepartum and postpartum periods (*BWS* = *2.73 *±* 0.14* vs. *2.45 *±* 0.18*,* p* = 0.19).

**Table 4:** Individual best–worst scaling scores for 11 statements in the antepartum versus postpartum periods among 154 HIV‐positive pregnant women

Statement	Antepartum		Postpartum		Difference		*p*‐value^a^
	Mean	SE	Mean	SE	Mean	SE	
I trust that doctors and nurses know what is best for infant's health	7.34	0.13	7.21	0.11	0.12	0.17	0.46
I can live as long as someone without HIV if I take care of myself	6.77	0.13	6.86	0.10	−0.09	0.16	0.56
Medications I take prevent my infant to get infected or become sick	6.11	0.12	6.54	0.09	−0.43	0.15	0.01
Medications to prevent disease help me feel stronger	6.09	0.10	5.83	0.07	0.26	0.13	0.05
I know the purpose of each different medication I take	6.08	0.11	6.35	0.10	−0.27	0.14	0.06
I have trouble taking medication on a daily basis	4.43	0.10	5.03	0.11	−0.59	0.14	<0.01
Friends and families help me to take medications	4.17	0.17	3.89	0.17	0.27	0.23	0.23
I am too busy to come to regular clinic visits	4.01	0.12	4.45	0.14	−0.44	0.17	0.01
Getting to the clinic costs me too much money.	3.90	0.11	3.52	0.09	0.38	0.14	0.01
I worry that if I take pills it can cause side effects on my infant	3.36	0.12	2.84	0.10	0.52	0.16	<0.01
I worry that taking pills every day tells other people that I have HIV	2.73	0.14	2.45	0.18	0.28	0.21	0.19

a *p*−values were calculated from Wald tests comparing mean BWS scores by timing of visits.

### 3.5 Association between changes in individual BWS scores and other factors

Several factors were examined for the association with changes in the individual BWS scores in the antepartum versus postpartum periods using multiple linear regressions. IPT initiation at enrolment was associated with significantly higher individual BWS scores for prevention of infant illness (β = 0.99 ± 0.34, *p* = 0.01) after adjusting for perceived risk of TB in next year. IPT initiation was also associated with the decrease in the BWS scores for side effects for infants (β = −1.19 ± 0.35, *p* < 0.01). Being adherent to ART was significantly associated with higher BWS scores for living a long life (*β* = 1.42 ± 0.65, *p* = 0.03) after adjusting for IPT initiation.

## 4 Discussion

Pregnant women newly diagnosed with HIV are faced with the decision to commit to lifelong ART, and this decision is often based on the health of their infants. Our study findings show that pregnant women with a recent HIV diagnosis who opt for receiving lifelong ART prioritized their trust in healthcare providers and prevention of infant illness most highly and these preferences persisted after delivery. Women also continued to highly prioritize having a long life and the benefit of medication for their own health compared to other structural barriers. However, the perceived difficulty in adherence and lack of time to attend regular clinic visits were more significant in the postpartum period. These reflect the complicated challenges mothers face after delivery and provide important insights to design and deliver effective interventions to increase uptake of ART and IPT.

The fact that trust in healthcare providers was most highly prioritized indicates how much positive influence providers can have on maternal perception about preventive therapy and engagement in clinical care, similar to findings from other studies 15, 41, 42. Over 90% of our study participants reported receiving help from clinical staff to remember to take their medications, which is reassuring. A recent study among HIV‐positive women in Ethiopia and Mozambique has shown that respectful care by providers was the most important determinant for seeking continued care after delivery 42. We found that mothers also continued to highly prioritize medication benefits for their own health in the postpartum period, almost two times higher than they did for structural barriers. Furthermore, knowledge about the purpose of different medications and adherence to ART were significantly associated with the higher BWS score for living a long life in the postpartum period.

Earlier studies documented that HIV‐positive women were less likely to be motivated to seek care in the postpartum period once they had protected their infants from HIV transmission, largely due to the demands of everyday life coupled with a lack of understanding of the benefits of therapy for their own health 10, 12, 43, 44. Other studies have shown that receiving sufficient information about ART or IPT was associated with better adherence and retention in care among HIV‐positive postpartum women 15, 41 as well as among HIV‐positive patients in general 45, 46, 47. A recent study among HIV‐positive pregnant women in South Africa showed that health education or counselling in clinics was perceived as more useful for linkage to and retention in care compared to financial incentives or home visits, as similarly shown among adults newly diagnosed with HIV in Mozambique 48, 49. Our study findings confirm that interventions focusing on building trust with healthcare providers and patient education about medication benefits can be highly effective in gaining patients’ utility and potentially encourage the uptake of and adherence to preventive therapy 47.

Almost half of participants received no such support despite the perception that family was an important source of support to remain in care in our study setting 23. About 20% were somewhat or very dissatisfied with the treatment support they received from friends and family and this corresponds to our finding that support from friends and family was only moderately ranked in both antepartum and postpartum periods. Having a good support system is vital for retention in care and adherence to ART and IPT among HIV‐positive women 15, 30, 50. Interventions to involve partners such as accompanying women to clinics or participating in couples counselling have been reported to increase ART adherence 51, 52. More studies are needed to effectively inform and engage family members and partners to support HIV‐positive pregnant and postpartum women.

It may seem contradictory that the burden of transportation costs, lack of time and the stigma surrounding taking daily medication were least prioritized in this study, compared to other studies where these were identified as major barriers for uptake of ART and IPT 31, 46, 53. At least three considerations may factor into this counterintuitive result. One, half of the participants in our study walked to their clinic visits; thus, the cost of transportation was often low or nonexistent. Two, recommended clinic visits for pregnant women are aligned with monthly ART pick‐up in South Africa, such that any additional transport cost, inconvenience, and stigma related to preventive therapy may have been small. Three, women may not have been concerned with pill burden because nearly all (98%) of our study population were receiving their antiretroviral therapy as one pill of fixed‐dose combination tablets 12, 54. As most of our participants had already disclosed their HIV status to their partners, any concern about unintended disclosures was moderated. The lack of time to regularly attend clinic visits, however, was more prioritized in the postpartum period and more participants reported that they had trouble taking medication on a daily basis at this stage. These problems potentially reflect the life‐changing impact of having and caring for a newborn baby and suggest that improved intervention and additional support for postpartum women are required.

In accord with the findings of other studies and reports, IPT prescription at enrolment was low at around 30%, and adherence to IPT was sub‐optimal, especially when measured using urine samples, although our sample size is too small 55. Compared to those on ART, fewer individuals on IPT indicated that staff had informed them of the purpose or side effects of IPT at their last clinic visits. While overall prioritization of attributes regarding preventive therapy did not differ by receiving IPT or not, those who had initiated IPT during pregnancy were significantly more likely to perceive the benefits of medications on infant's health in the postpartum period. To increase uptake of IPT and ensure better adherence, efforts to enhance the perception of its benefits for both maternal and infants’ health are warranted.

There are several limitations to this study. Firstly, recruitment and follow‐up of participants were logistically difficult. Based on the District Health Information System data in South Africa, we estimate about 40% of all eligible women could not be recruited for this study, despite vigorous efforts to do so 56. We used three interviewers to rotate through all 14 clinics for recruitment, which limited our capacity to enroll all eligible women presenting at ANC visits. Although we tracked down study participants who missed scheduled study visits, the completion of follow‐up visits was sub‐optimal and the timing of postpartum visits varied. We cannot ensure whether those lost to follow up may have had different priorities regarding health or structural barriers linked to care. However, the baseline characteristics did not differ between those lost to follow up and retained in care nor by the timing of completed postpartum visits. Secondly, we asked participants to choose between statements with both positive and negative connotations in the same choice task. Our results, therefore, can only infer which statements were perceived as best, and which were perceived as worst. However, we saw that the absolute rankings of preferences remained similar in the postpartum period, supporting the belief that our instrument likely had good face validity. Future efforts could expand on this work by elucidating the degree to which positive statements refer to facilitators of preventive therapy, and negative statements refer to barriers. Thirdly, we could not verify the validity of self‐reported adherence for ART against other objective methods. However, we used a short recall period shown to have good association with more objective measures.

## 5 Conclusions

We have quantified maternal priorities for preventive therapy among HIV‐positive pregnant women around the time of delivery using BWS. We demonstrated that trust in healthcare providers and medication benefits for both infants’ and their own health were consistently most highly prioritized in the antepartum and postpartum periods. While it is important to address structural barriers, comprehensive interventions should focus on increasing support from healthcare providers and family members and improving women's knowledge on medication benefits of ART and IPT. Such interventions could be highly accepted and more effective to encourage uptake of preventive therapy with an ultimate goal of improving the health of HIV‐positive mothers and their infants in high HIV and TB burden settings.

## Competing interests

The authors have no conflicts to declare.

## Authors’ contribution

All authors contributed to design the study. H‐YK, CFH and NM helped to collect the data. JFPB and H‐YK contributed to analyse the data. H‐YK wrote the initial draft of the paper. All authors contributed to read and revised the final manuscript.

## Supporting information


**Figure S1.** Flow chart for study participants at six weeks, 14 week and later than 14 weeks post‐partum visits.
**Table S1.** Participants’ characteristics by the timing of postpartum visits included in the main analysis (N = 154)
**Table S2.** Aggregate BWS scores in the antepartum versus postpartum periods by the timing of postpartum visits (N = 154)
**Table S3.1.** Individual best–worst scaling scores for 11 statements in the antepartum versus postpartum periods among participants who completed the survey at six weeks postpartum visits and were included in the main analysis (N = 33)
**Table S3.2.** Individual best–worst scaling scores for 11 statements in the antepartum versus postpartum periods among all participants who completed the survey at 6 weeks postpartum visits (N = 93)
**Table S3.3.** Individual best–worst scaling scores for 11 statements in the antepartum versus postpartum periods among participants who completed the survey at 14 weeks postpartum visits included in the main analysis (N = 77)
**Table S3.4.** Individual best–worst scaling scores for 11 statements in the antepartum versus postpartum periods among participants who completed the survey at >14 weeks postpartum visits and were included in the main analysis (N = 44)
**Figure S2.** Changes in individual best–worst scaling (BWS) scores for 11 statements related to preventive therapies among HIV‐positive pregnant women in the postpartum versus antepartum periods.
**Figure S3.** Full list of 11 questions included in the questionnaire.
