# Supplementary material for: Maternal priorities for preventive therapy among HIV‐positive pregnant women before and after delivery in South Africa: a best–worst scaling survey
Source: J Int AIDS Soc. 2018 Jul 4;21(7):e25143. doi: 10.1002/jia2.25143 (PMC6031072; doi:10.1002/jia2.25143)
Supplement: Supplementary file 1 — Figure S1. Flow chart for study participants at six weeks, 14 week and later than 14 weeks post‐partum visits. Table S1. Participants’ characteristics by the timing of postpartum visits included in the main analysis (N = 154) Table S2. Aggregate BWS scores in the antepartum versus postpartum periods by the timing of postpartum visits (N = 154) Table S3.1. Individual best–worst scaling scores for 11 statements in the antepartum versus postpartum periods among participants who completed the survey at six weeks postpartum visits and were included in the main analysis (N = 33) Table S3.2. Individual best–worst scaling scores for 11 statements in the antepartum versus postpartum periods among all participants who completed the survey at 6 weeks postpartum visits (N = 93) Table S3.3. Individual best–worst scaling scores for 11 statements in the antepartum versus postpartum periods among participants who completed the survey at 14 weeks postpartum visits included in the main analysis (N = 77) Table S3.4. Individual best–worst scaling scores for 11 statements in the antepartum versus postpartum periods among participants who completed the survey at >14 weeks postpartum visits and were included in the main analysis (N = 44) Figure S2. Changes in individual best–worst scaling (BWS) scores for 11 statements related to preventive therapies among HIV‐positive pregnant women in the postpartum versus antepartum periods. Figure S3. Full list of 11 questions included in the questionnaire. [file JIA2-21-e25143-s001.docx]

**Maternal priorities for preventive therapy among HIV-positive pregnant women before and after delivery in South Africa: a best-worst scaling survey**

**SUPPLEMENT**

N=204

Enrolled participants

n=111

- Relocation (n=15)

- Missed 6 weeks visit^*^ (n=63)

- Lost to follow up^†^ (n=24)

- Stillbirth (n=1)

- Miscarriage (n=2)

- Refused to follow up^‡^ (n=6)

n=93 (45.6%)

6 weeks postpartum visit

n=28

Missed 14 weeks visit but completed visit at >14 weeks

n=77 (37.8%)

14 weeks postpartum visit

n=12

Completed visit at 14 weeks

n=35

Completed visit at >14 weeks

n=63 (30.9%)

postpartum visit at > 14 weeks

**Figure S1. Flow chart for study participants at 6 weeks, 14 week and later than 14 weeks post-partum visits.**

*Missed visit refers to either a client did not come for the visit or our study staff missed her appointed visit.

†If a study participant could not be reached by phone more than three times, she was considered as lost to follow up.

‡Some refused to be followed or were no longer interested in participating in the study.

**Table S1.** Participants’ characteristics by the timing of postpartum visits included in the main analysis (N=154)

| ***N (%)*** | **6 weeks**  **(N=33)** | **14 weeks (N=77)** | **>14 weeks (N=44)** | **p-value** |
| --- | --- | --- | --- | --- |
| **Age (years),** Mean (± SD) | 27 (± 6) | 27 (± 6) | 28(± 6) | 0.92 |
| **Gestational week at first ANC visit,** | 21 (± 7) | 18 (± 8) | 17 (± 8) | 0.02 |
| Mean (± SD) |  |  |  |  |
| **Gestational week at enrollment,**  Mean (± SD) | 29 (± 8) | 23 (± 9) | 24 (± 8) | 0.01 |
| **Time since HIV diagnosis (days),**  Mean (± SD) | 63 (± 44) | 52 (± 49) | 52 (± 63) | 0.52 |
| **CD4 cell count (cells/mm^3^),**  Mean (± SD)**^**^** | 493 (±262) | 418 (±204) | 532 (±268) | 0.09 |
| **Time since delivery (weeks)** |  |  |  |  |
| Mean ± SD | 8.9 ± 3.5 | 17.2 ± 3.8 | 33.2 ± 9.7 | N/A |
| Median (Q1, Q3) | 7 (6, 11) | 16 (15, 19) | 32 (26, 40) |  |
| **Perceived risk of developing TB within next year^***^** |  |  |  |  |
| Slightly likely | 5 (15.6) | 17 (22.1) | 6 (13.6) | 0.47 |
| It is not at all likely | 27 (84.4) | 60 (77.9) | 38 (86.4) |  |
| **Education^**^** |  |  |  |  |
| ≤ 9 grade | 4 (16.7) | 13 (23.2) | 13 (38.2) | 0.14 |
| > 9 grade | 20 (83.3) | 43 (76.8) | 21 (61.8) |  |
| **Employment status** |  |  |  |  |
| Full time | 3 ( 9.1) | 5 ( 6.5) | 5 (11.4) | 0.59 |
| Part time or piece jobs | 1 ( 3.0) | 6 ( 7.8) | 5 (11.4) |  |
| Unemployed | 29 (87.9) | 66 (85.7) | 34 (77.3) |  |
| **Mode of transportation to clinic** |  |  |  |  |
| On foot | 18 (54.5) | 45 (58.4) | 21 (47.7) | 0.51 |
| Public taxi or bus | 15 (45.5) | 31 (40.3) | 21 (47.7) |  |
| Private car or motorbike | 0 ( 0.0) | 1 ( 1.3) | 2 ( 4.5) |  |
| **Transportation cost (Rand)** |  |  |  |  |
| Median (IQR) | 0 (0, 16) | 0 (0, 16) | 0 (0, 18) | 0.89 |
| **Marital status** |  |  |  |  |
| Married | 6 (18.2) | 5 ( 6.7) | 6 (14.0) | 0.27 |
| Living with partner | 6 (18.2) | 30 (40.0) | 16 (37.2) |  |
| Not living with partner | 21 (63.6) | 40 (53.3) | 21 (48.8) |  |
| **Disclosure of HIV status to partner** |  |  |  |  |
| Yes | 26 (81.3) | 65 (86.7) | 34 (81.0) | 0.65 |
| No | 6 (18.8) | 10 (13.3) | 8 (19.0) |  |

**Table S2. Aggregate BWS scores in the antepartum vs. postpartum periods by the timing of postpartum visits (N=154)**

|  | Antepartum | Postpartum | | | |  |
| --- | --- | --- | --- | --- | --- | --- |
| **Statements** |  | All | 6 weeks | 14 weeks | >14 weeks | 6 weeks* |
|  | N=154 | N=154 | N=33 | N=77 | N=44 | N=93 |
|  | Mean ± SE | Mean ± SE | Mean ± SE | Mean ± SE | Mean ± SE | Mean ± SE |
| I trust that doctors and nurses know what is best for infant's health. | 73.4 ± 1.0 | 72.1 ± 1.0 | 73.9 ± 2.1 | 73.4 ± 1.4 | 68.6 ± 1.9 | 72.9 ± 2.1 |
| I can live as long as someone without HIV if I take care of myself. | 67.7 ± 1.1 | 68.6 ± 1.0 | 68.5 ± 2.3 | 67.9 ± 1.3 | 70.0 ± 1.8 | 67.4 ± 2.1 |
| Medications I take prevent my infant to get infected or become sick. | 61.1 ± 1.1 | 65.4 ± 1.0 | 59.7 ± 2.1 | 67.4 ± 1.4 | 66.1 ± 1.7 | 62.5 ± 2.1 |
| Medications to prevent disease help me feel stronger. | 60.9 ± 1.0 | 58.3 ± 0.8 | 58.5 ± 1.8 | 58.8 ± 1.1 | 57.3 ± 1.5 | 60.9 ± 1.9 |
| I know the purpose of each different medication I take. | 60.8 ± 0.9 | 63.5 ± 0.9 | 61.5 ± 2.2 | 66.5 ± 1.2 | 59.8 ± 1.5 | 60.2 ± 2.2 |
| I have trouble taking medication on a daily basis. | 44.3 ± 1.0 | 50.3 ± 0.9 | 48.8 ± 2.0 | 50.3 ± 1.3 | 51.4 ± 1.7 | 48.6 ± 2.0 |
| Friends and families help me to take medications. | 41.7 ± 1.2 | 38.9 ± 1.2 | 43.9 ± 2.6 | 37.0 ± 1.7 | 38.4 ± 2.2 | 44.3 ± 2.6 |
| I am too busy to come to regular clinic visits. | 40.1 ± 0.9 | 44.5 ± 0.9 | 40.9 ± 2.1 | 43.6 ± 1.4 | 48.6 ± 1.7 | 41.4 ± 2.1 |
| Getting to the clinic costs me too much money. | 39.0 ± 0.9 | 35.2 ± 0.9 | 32.4 ± 2.1 | 35.1 ± 1.2 | 37.5 ± 1.7 | 34.8 ± 1.9 |
| I worry that if I take pills it can cause side effects on my infant. | 33.6 ± 1 | 28.4 ± 1.0 | 32.1 ± 2.3 | 26.1 ± 1.3 | 29.5 ± 1.8 | 30.8 ± 2.2 |
| I worry that taking pills every day tells other people that I have HIV. | 27.3 ± 1 | 24.5 ± 1.1 | 29.1 ± 2.4 | 23.8 ± 1.5 | 22.5 ± 2.0 | 25.9 ± 2.2 |

*****Data include participants who completed both 6 weeks visits and 14 weeks visits thus only their 14 weeks visits were included for the main analysis.

**Table S3.1 Individual Best-Worst Scaling scores for 11 statements in the antepartum vs. postpartum periods among participants who completed the survey at 6 weeks postpartum visits and were included in the main analysis (N=33)**

| Statement | Antepartum | | Postpartum | | Difference | |  |
| --- | --- | --- | --- | --- | --- | --- | --- |
|  | Mean | SE | Mean | SE | Mean | SE | p-value |
| I have trouble taking medication on a daily basis. | 4.52 | 0.29 | 4.88 | 0.18 | 0.36 | 0.34 | 0.30 |
| I am too busy to come to regular clinic visits. | 3.85 | 0.21 | 4.09 | 0.31 | 0.24 | 0.38 | 0.53 |
| Medications I take prevent my infant to get infected or become sick. | 6.42 | 0.29 | 5.97 | 0.18 | -0.45 | 0.30 | 0.14 |
| I know the purpose of each different medication I take. | 6.12 | 0.24 | 6.15 | 0.28 | 0.03 | 0.36 | 0.93 |
| I can live as long as someone without HIV if I take care of myself. | 6.58 | 0.34 | 6.85 | 0.23 | 0.27 | 0.40 | 0.50 |
| I trust that doctors and nurses know what is best for infant's health. | 7.00 | 0.29 | 7.39 | 0.25 | 0.39 | 0.38 | 0.31 |
| Medications to prevent disease help me feel stronger. | 6.18 | 0.22 | 5.85 | 0.14 | -0.33 | 0.30 | 0.27 |
| Friends and families help me to take medications. | 4.82 | 0.43 | 4.39 | 0.39 | -0.42 | 0.59 | 0.48 |
| I worry that taking pills every day tells other people that I have HIV. | 2.73 | 0.31 | 2.91 | 0.33 | 0.18 | 0.44 | 0.68 |
| Getting to the clinic costs me too much money. | 3.42 | 0.28 | 3.24 | 0.24 | -0.18 | 0.30 | 0.55 |
| I worry that if I take pills it can cause side effects on my infant. | 3.33 | 0.22 | 3.21 | 0.26 | -0.12 | 0.31 | 0.70 |

**Table S3.2 Individual Best-Worst Scaling scores for 11 statements in the antepartum vs. postpartum periods among all participants who completed the survey at 6 weeks postpartum visits (N=93)***

| Statement | Antepartum | | Postpartum | | Difference | |  |
| --- | --- | --- | --- | --- | --- | --- | --- |
|  | Mean | SE | Mean | SE | Mean | SE | p-value |
| I have trouble taking medication on a daily basis. | 4.41 | 0.13 | 4.86 | 0.13 | -0.45 | 0.19 | 0.02 |
| I am too busy to come to regular clinic visits. | 4.03 | 0.16 | 4.14 | 0.17 | -0.11 | 0.23 | 0.64 |
| Medications I take prevent my infant to get infected or become sick. | 6.14 | 0.15 | 6.25 | 0.13 | -0.11 | 0.19 | 0.57 |
| I know the purpose of each different medication I take. | 6.15 | 0.14 | 6.02 | 0.17 | 0.13 | 0.20 | 0.53 |
| I can live as long as someone without HIV if I take care of myself. | 6.74 | 0.17 | 6.74 | 0.13 | 0.00 | 0.22 | 1.0 |
| I trust that doctors and nurses know what is best for infant's health. | 7.08 | 0.17 | 7.29 | 0.15 | -0.22 | 0.21 | 0.3 |
| Medications to prevent disease help me feel stronger. | 6.13 | 0.13 | 6.09 | 0.11 | 0.04 | 0.19 | 0.82 |
| Friends and families help me to take medications. | 4.35 | 0.23 | 4.43 | 0.22 | -0.08 | 0.32 | 0.82 |
| I worry that taking pills every day tells other people that I have HIV. | 2.71 | 0.18 | 2.59 | 0.19 | 0.12 | 0.25 | 0.64 |
| Getting to the clinic costs me too much money. | 3.8 | 0.14 | 3.48 | 0.13 | 0.31 | 0.18 | 0.08 |
| I worry that if I take pills it can cause side effects on my infant. | 3.44 | 0.15 | 3.08 | 0.15 | 0.37 | 0.20 | 0.07 |

*****Data include participants who completed both 6 weeks visits and 14 weeks visits thus only their 14 weeks visits were o included for the main analysis.

**Table S3.3 Individual Best-Worst Scaling scores for 11 statements in the antepartum vs. postpartum periods among participants who completed the survey at 14 weeks postpartum visits included in the main analysis (N=77)**

| Statement | Antepartum | | Postpartum | | Difference | |  |
| --- | --- | --- | --- | --- | --- | --- | --- |
|  | Mean | SE | Mean | SE | Mean | SE | p-value |
| I have trouble taking medication on a daily basis. | 4.3 | 0.11 | 5.03 | 0.16 | 0.73 | 0.20 | <0.01 |
| I am too busy to come to regular clinic visits. | 4.06 | 0.18 | 4.36 | 0.20 | 0.3 | 0.24 | 0.21 |
| Medications I take prevent my infant to get infected or become sick. | 6.08 | 0.15 | 6.74 | 0.14 | 0.66 | 0.21 | <0.01 |
| I know the purpose of each different medication I take. | 6.12 | 0.15 | 6.65 | 0.13 | 0.53 | 0.20 | 0.01 |
| I can live as long as someone without HIV if I take care of myself. | 6.78 | 0.18 | 6.79 | 0.12 | 0.01 | 0.21 | 0.95 |
| I trust that doctors and nurses know what is best for infant's health. | 7.29 | 0.18 | 7.34 | 0.14 | 0.05 | 0.23 | 0.82 |
| Medications to prevent disease help me feel stronger. | 6.17 | 0.14 | 5.88 | 0.11 | -0.29 | 0.19 | 0.14 |
| Friends and families help me to take medications. | 3.88 | 0.24 | 3.70 | 0.23 | -0.18 | 0.32 | 0.57 |
| I worry that taking pills every day tells other people that I have HIV. | 2.90 | 0.20 | 2.38 | 0.26 | -0.52 | 0.30 | 0.09 |
| Getting to the clinic costs me too much money. | 3.90 | 0.14 | 3.51 | 0.11 | -0.39 | 0.17 | 0.02 |
| I worry that if I take pills it can cause side effects on my infant. | 3.51 | 0.17 | 2.61 | 0.13 | -0.90 | 0.23 | <0.01 |

**Table S3.4 Individual Best-Worst Scaling scores for 11 statements in the antepartum vs. postpartum periods among participants who completed the survey at > 14 weeks postpartum visits and were included in the main analysis (N=44)**

| Statement | Antepartum | | Postpartum | | Difference | |  |
| --- | --- | --- | --- | --- | --- | --- | --- |
|  | Mean | SE | Mean | SE | Mean | SE | p-value |
| I have trouble taking medication on a daily basis. | 4.59 | 0.18 | 5.14 | 0.19 | 0.55 | 0.24 | 0.03 |
| I am too busy to come to regular clinic visits. | 4.05 | 0.22 | 4.86 | 0.23 | 0.82 | 0.34 | 0.02 |
| Medications I take prevent my infant to get infected or become sick. | 5.93 | 0.22 | 6.61 | 0.16 | 0.68 | 0.28 | 0.02 |
| I know the purpose of each different medication I take. | 6.00 | 0.19 | 5.98 | 0.14 | -0.02 | 0.23 | 0.92 |
| I can live as long as someone without HIV if I take care of myself. | 6.91 | 0.23 | 7.00 | 0.19 | 0.09 | 0.28 | 0.75 |
| I trust that doctors and nurses know what is best for infant's health. | 7.68 | 0.24 | 6.86 | 0.22 | -0.82 | 0.20 | <0.01 |
| Medications to prevent disease help me feel stronger. | 5.89 | 0.20 | 5.73 | 0.14 | -0.16 | 0.24 | 0.51 |
| Friends and families help me to take medications. | 4.18 | 0.29 | 3.84 | 0.30 | -0.34 | 0.41 | 0.41 |
| I worry that taking pills every day tells other people that I have HIV. | 2.45 | 0.23 | 2.25 | 0.34 | -0.2 | 0.41 | 0.62 |
| Getting to the clinic costs me too much money. | 4.25 | 0.21 | 3.75 | 0.17 | -0.5 | 0.30 | 0.10 |
| I worry that if I take pills it can cause side effects on my infant. | 3.11 | 0.24 | 2.95 | 0.21 | -0.16 | 0.33 | 0.63 |

**
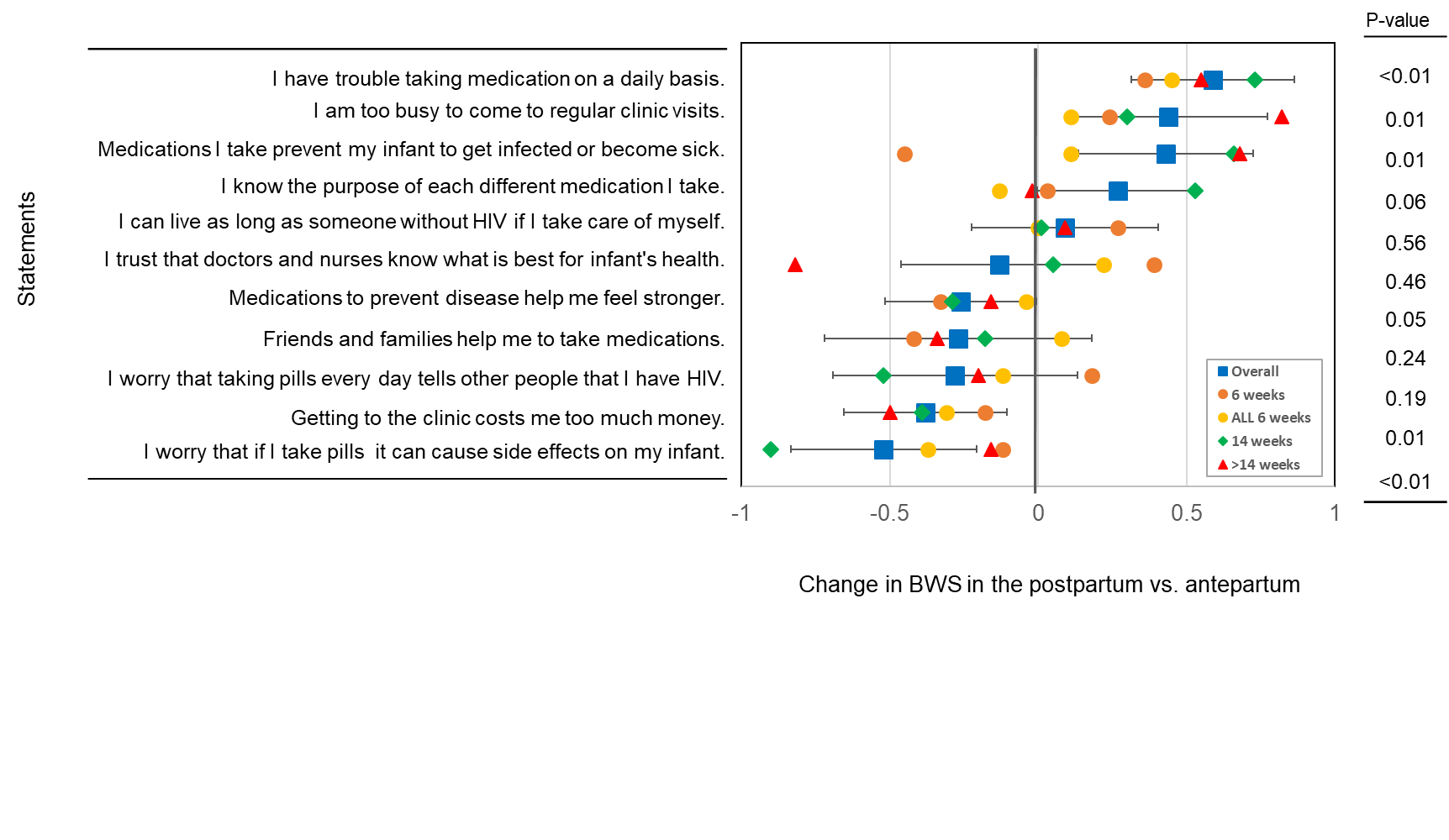
**

**Figure S2**. Changes in individual Best-Worst Scaling (BWS) scores for 11 statements related to preventive therapies among HIV-positive pregnant women in the postpartum vs. antepartum periods. Error bars show 95% confidence intervals, and p-values from Wald tests comparing mean BWS scores in the postpartum vs. antepartum periods are presented to the right. Square represents data from all participants (N=154); orange circle from participants at 6 weeks visits included in the main analysis (N=33); yellow circle from all participants who completed 6 weeks visits (N=93); diamond from participants at 14 weeks visits (N=77); triangle from participants at >14 weeks visits (N=44).

**Figure S3. Full list of 11 questions included in the questionnaire**

| Now I’m going to show you some sets of 5 statements. For each set, choose the one that BEST describes your thought (or the statement you agree with the most), and the one that WORST describes your thought (the statement you disagree with the most). Each new set will have some different statements, and some will be repeated.  Take your time and look over each statement. Remember, there are no right or wrong answers. | | | |
| --- | --- | --- | --- |
|  |  | **Best** | **Worst** |
|  | A. Friends and families help me to take medications. | ⃝ | ⃝ |
|  | B. I worry that taking pills every day tells other people that I have HIV. | ⃝ | ⃝ |
|  | C. Medications I take prevent my infant to get infected or become sick. | ⃝ | ⃝ |
|  | D. Getting to the clinic costs me too much money. | ⃝ | ⃝ |
|  | E. I trust that doctors and nurses know what is best for infant's health. | ⃝ | ⃝ |
|  |  | **Best** | **Worst** |
|  | A. I know the purpose of each different medication. | ⃝ | ⃝ |
|  | B. I trust that doctors and nurses know what is best for infant's health. | ⃝ | ⃝ |
|  | C. I worry that taking pills every day tells other people that I have HIV. | ⃝ | ⃝ |
|  | D. I can live as long as someone without HIV if I take care of myself. | ⃝ | ⃝ |
|  | E. I have trouble taking medication on a daily basis. | ⃝ | ⃝ |
|  |  | **Best** | **Worst** |
|  | A. I can live as long as someone without HIV if I take care of myself. | ⃝ | ⃝ |
|  | B. I know the purpose of each different medication. | ⃝ | ⃝ |
|  | C. Friends and families help me to take medications. | ⃝ | ⃝ |
|  | D. Medications I take prevent my infant to get infected or become sick. | ⃝ | ⃝ |
|  | E. I am too busy to come to regular clinic visits. | ⃝ | ⃝ |
|  |  | **Best** | **Worst** |
|  | A. Medications to prevent disease help me feel stronger. | ⃝ | ⃝ |
|  | B. I am too busy to come to regular clinic visits. | ⃝ | ⃝ |
|  | C. I know the purpose of each different medication. | ⃝ | ⃝ |
|  | D. I worry that taking pills every day tells other people that I have HIV. | ⃝ | ⃝ |
|  | E. Getting to the clinic costs me too much money. | ⃝ | ⃝ |
|  |  | **Best** | **Worst** |
|  | A. Medications I take prevent my infant to get infected or become sick. | ⃝ | ⃝ |
|  | B. I can live as long as someone without HIV if I take care of myself. | ⃝ | ⃝ |
|  | C. Getting to the clinic costs me too much money. | ⃝ | ⃝ |
|  | D. I have trouble taking medication on a daily basis. | ⃝ | ⃝ |
|  | E. Medications to prevent disease help me feel stronger | ⃝ | ⃝ |

|  |  | **Best** | **Worst** |
| --- | --- | --- | --- |
|  | A. I worry that if I take pills, it can cause side effects on my infant. | ⃝ | ⃝ |
|  | B. Getting to the clinic costs me too much money. | ⃝ | ⃝ |
|  | C. I am too busy to come to regular clinic visits. | ⃝ | ⃝ |
|  | D. I trust that doctors and nurses know what is best for infant's health. | ⃝ | ⃝ |
|  | E. I can live as long as someone without HIV if I take care of myself. | ⃝ | ⃝ |
|  |  |  |  |
|  | A. Getting to the clinic costs me too much money. | **Best** | **Worst** |
|  | B. Friends and families help me to take medications. | ⃝ | ⃝ |
|  | C. I have trouble taking medication on a daily basis. | ⃝ | ⃝ |
|  | D. I worry that if I take pills, it can cause side effects on my infant. | ⃝ | ⃝ |
|  | E. I know the purpose of each different medication. | ⃝ | ⃝ |
|  |  | **Best** | **Worst** |
|  | A. I am too busy to come to regular clinic visits. | ⃝ | ⃝ |
|  | B. I have trouble taking medication on a daily basis. | ⃝ | ⃝ |
|  | C. I trust that doctors and nurses know what is best for infant's health. | ⃝ | ⃝ |
|  | D. Medications to prevent disease help me feel stronger. | ⃝ | ⃝ |
|  | E. Friends and families help me to take medications. | ⃝ | ⃝ |
|  |  | **Best** | **Worst** |
|  | A. I have trouble taking medication on a daily basis. | ⃝ | ⃝ |
|  | B. Medications I take prevent my infant to get infected or become sick. | ⃝ | ⃝ |
|  | C. I worry that if I take pills, it can cause side effects on my infant. | ⃝ | ⃝ |
|  | D. I am too busy to come to regular clinic visits. | ⃝ | ⃝ |
|  | E. I worry that taking pills every day tells other people that I have HIV. | ⃝ | ⃝ |
|  |  | **Best** | **Worst** |
|  | A. I worry that taking pills every day tells other people that I have HIV. | ⃝ | ⃝ |
|  | B. Medications to prevent disease help me feel stronger. | ⃝ | ⃝ |
|  | C. I can live as long as someone without HIV if I take care of myself. | ⃝ | ⃝ |
|  | D. Friends and families help me to take medications. | ⃝ | ⃝ |
|  | E. I worry that if I take pills, it can cause side effects on my infant. | ⃝ | ⃝ |
|  |  | **Best** | **Worst** |
|  | A. I trust that doctors and nurses know what is best for infant's health. | ⃝ | ⃝ |
|  | B. I worry that if I take pills, it can cause side effects on my infant. | ⃝ | ⃝ |
|  | C. Medications to prevent disease help me feel stronger. | ⃝ | ⃝ |
|  | D. I know the purpose of each different medication. | ⃝ | ⃝ |
|  | E. Medications I take prevent my infant to get infected or become sick. | ⃝ | ⃝ |
